# Acute Myeloid Leukemia and the Bone Marrow Niche—Take a Closer Look

**DOI:** 10.3389/fonc.2018.00444

**Published:** 2018-10-12

**Authors:** Lena Behrmann, Jasmin Wellbrock, Walter Fiedler

**Affiliations:** Department of Oncology, Hematology and Bone Marrow Transplantation with Section Pneumology, Hubertus Wald University Cancer Center, University Medical Center Hamburg Eppendorf, Hamburg, Germany

**Keywords:** acute myeloid leukemia, AML, bone marrow, angiogenesis, niche, endothelial cell, vasculature, 3D confocal microscopy

## Abstract

The bone marrow is the home of hematopoiesis and is therefore a hotspot for the development of hematopoietic diseases. Complex interactions between the bone marrow microenvironment and hematopoietic stem cells must find a balance between proliferation, differentiation and homeostasis of the stem cell compartment. Changes in this tightly regulated network can provoke malignant transformation, leading to hematopoietic diseases. Here we focus on acute myeloid leukemia (AML), since this is the most frequent acute leukemia in adulthood with very poor overall survival rates and where relapse after chemotherapy continues to be a major challenge, driving demand for new therapeutic strategies. Current research is focusing on the identification of specific interactions between leukemic blasts and their niche components, which may be exploited as novel treatment targets along with induction chemotherapy. Significant progress has been gained over the last few years in the field of high-resolution imaging. Confocal *ex vivo* and intravital microscopy have revealed a detailed map of bone marrow structures and components; as well as identifying numerous alterations in the stem cell niche that correspond to disease progression. However, the underlying mechanisms are still not completely understood and due to the complexity, their elucidation remains a challenging. This review discusses the constitution of the AML niche in the bone marrow, the improvement in visualization of the complex three-dimensional niche structures and points out new therapeutic strategies to increase the overall survival of AML patients.

## Introduction to acute myeloid leukemia

Leukemia is characterized by infiltration of the hematopoietic organs (bone marrow, blood, spleen, and other tissues) by abnormally differentiated and nonfunctional hematopoietic blasts (1). High and uncontrolled proliferation of leukemic cells causes the expulsion of the normal hematopoietic system and the loss of their functions, leading to life-threatening symptoms such as thrombocytopenia, anemia, and immunodeficiency. Although the clinical presentation of the disease is quite uniform, the genetic and cytogenetic landscape of leukemia's is very heterogeneous (1–3). Proximal causes of leukemic development can be categorized in three major groups: (1) gene mutations and translocations, including epigenetic dysregulation, (2) immune dysregulation, and (3) changes in the bone marrow microenvironment.

Adult and elderly patients suffer mostly from the aggressive acute myeloid leukemia (AML) that shows an increasing incidence with age. Without therapy this disease leads to the death of the patient within a few weeks. Although the overall 5-year survival rate of AML patients improved by application of chemotherapy, it remains unsatisfactory. A positive outcome declines from 50% for those younger than age 65 years to only 23% for patients aged 65 years and higher (4). Although AML is known to be genetically and cytogenetically heterogeneous, the common clinical chemotherapy has not changed in the last three decades, consisting of sequential courses of cytarabine (AraC) and daunorubicine (7 + 3 regimen) (5). Particularly problematic in patients <60 years is a diminished tolerance for intensive chemotherapy with increased risk of treatment-related toxicity, leading to an estimated treatment-related mortality ranging from 10 to 30% (6). The second disadvantage of this non-specific treatment is a high rate of resistance and relapse. Disease recurrence occurs in 60–80% of AML patients within 3 years after diagnosis even in patients who achieved remission with chemotherapy.

AML is a multi-gene disease with complex subclonal populations. After chemotherapy leukemic clones are selected to those capable of activating resistance mechanisms, leading to relapsed, and refractory disease (7). Therefore, there is an urgent need for low-intensity targeted treatment to improve survival of AML patients. Over the last few years an evolving number of studies have deciphered the essential role of the microenvironment in the pathogenesis of AML. The understanding of the complex interactions between leukemia and its niche cells may reveal new drugable targets to improve AML therapy. This review will summarize the recent advances in elucidating the leukemic microenvironment in the bone marrow to decipher new perspectives for targeted therapies.

## Bone marrow microenvironment of leukemic cells

To understand the reasons for the emergence of refractory AML cells under and after chemotherapy we have to understand how resistance can develop. Resistance can be divided into the categories *de novo* and acquired: where the latter is a sequential genetic change that finally ends in prosurvival and antiapoptotic phenotypes (8). *De novo* drug resistance refers to the result of environment-mediated protection from apoptosis that enables resistant AML cells to survive. The most relevant microenvironment for AML is within the bone marrow, the organ that hosts the hematopoietic stem cells (HSCs) and facilitates their differentiation into the manifold blood cell lineages (9). Within the bone marrow the HSCs are located in a tightly controlled local microenvironment, the so called niche, that regulates self-renewal, quiescence, proliferation, and differentiation of HSCs by bound or secreted molecules emanated by the surrounding cells (10). In the past decades various cell types were implicated for their roles in promoting HSC maintenance, including osteoblasts, perivascular stromal cells, endothelial cells, macrophages, CXCL12-abundant reticular cells (CAR cells), sympathetic neurons and nonmyelinating Schwann cells (11–17). Additionally there are several known soluble factors relevant for HSCs, including CXCL12, angiopoietin 1 (ANGPT1), TGF-β and signaling pathways including Notch and Wnt (11, 18–22).

Advances in bone marrow imaging technologies have improved the understanding of the physical HSC niche localization and physiological architecture but there is still the potential for discovering other undetected cell populations and factors. Despite the complexity of the bone marrow microenvironment, the HSC niche can be reduced into two physical geographies, the endosteal, and perivascular niche. The endosteum is defined by immediate proximity to trabecular or cortical bone with a high content of osteoblasts (23). The perivascular niche is located in proximity to sinusoidal and arteriolar vascular endothelium, including the surrounding supportive structures such as stromal cells and extracellular matrix (24, 25). Many publications discuss the controversial role of the distinct niches in regard to HSC dormancy and maintenance (25–27). However, the endosteal niche is consistently intertwined with vascular structures, first at the sites where arterioles enter the bone marrow via the endosteal zone, and second at the sites of sinusoids that spread as a dense network through the entire bone marrow cavity (28). In reality a separation of the two microenvironments is difficult and HSCs interact with numerous and simultaneous cell-extrinsic signaling settings (29).

## Components of the AML niche

**Figure 1:**
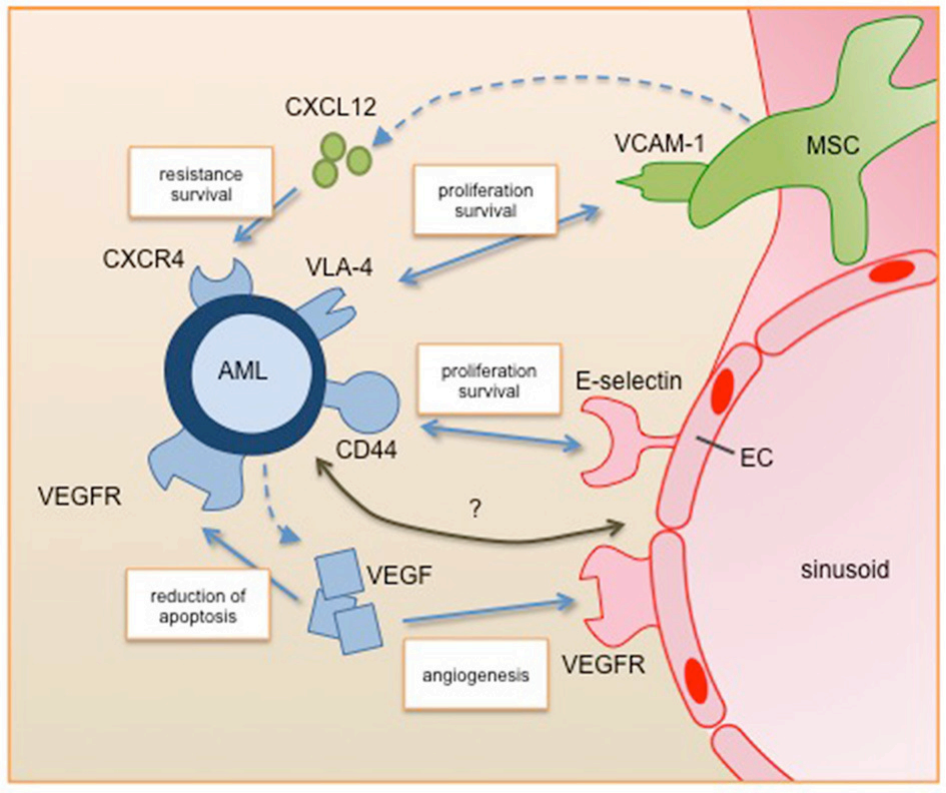
Leukemic blast interactions with the perivascular niche. Secreted factors and cell-cell interactions regulate the survival, proliferation and resistance of AML cells in the perivascular niche. All of them are under clinical investigation for targeted therapy. Future studies will identify further relevant pathways of the interplay between AML and its niche cells (marked with question mark). AML, acute myeloid leukemia; EC, endothelial cell; MSC, mesenchymal stromal cell; VEGF, vascular endothelial growth factor; VEGFR, vascular endothelial growth factor receptor, CXCL12, C-X-C motif chemokine 12; CXCR4, C-X-C chemokine receptor 4; VLA-4, very late antigen 4; VCAM-1, vascular cell adhesion molecule 1.

Like the hematopoietic system, AML is depicted as a hierarchical disease based on a small subset of leukemia initiating cells (LICs). This stem cell-like compartment has alone long-term repopulating potential, the ability to propagate and maintain the AML phenotype, and is expected to be the main cause for AML relapse (30). First LICs were identified as rare CD34^+^CD38^−^ events, as demonstrated by their capacity for serial transplantations in a mouse xenograft model (31, 32). As few as one in a million AML cells show this type of leukemia initiating activity, endorsing the idea of the hierarchical organization of the disease. Over the last years, an evolving number of further surface markers were defined, like CD123 or CD96, and even in the CD34^−^ fraction LICs can reside (33–35). Hence, the heterogeneous genetic landscape of AML is recapitulated within the stem cell phenotype. Independent of their phenotype, LICs have the potential to infiltrate into the HSC niche and hijack the normal homeostatic processes, supporting their self-renewal and proliferation potential, as well as quiescence and resistance to chemotherapy. Recent findings indicate that myeloid malignancies also alter the HSC niche into a leukemia niche that becomes permissive of leukemia growth and disrupts normal hematopoiesis (36). The corrupted components of the leukemic niche cooperate with LICs to maintain their quiescence and survival. Furthermore it has been suggested that mutations in stromal cells have a primary role in AML initiation (37). However, leukemia is a very heterogeneous disease and even though we have made gains in understanding the HSC niche, not all niche related pathways are relevant for AML or might even have an inverted effect for leukemic propagation. Summarizing the AML niche needs a careful discrimination between the different concepts of niches for HSC and other leukemic subtypes. Figure 1 summarizes drugable pathways for targeted AML therapy, which will be discussed in the following paragraphs.

A number of studies have confirmed that there is a tight relationship between leukemic and endothelial cells. For instance Cogle et al. presented that vascular endothelial cells are a critical component of the AML niche and leukemic cells can integrate into the vasculature (38). Furthermore, the two-way communication between AML and endothelial cells through autocrine and paracrine pathways supports AML development and progression. Here, a key player is the proangiogenic factor VEGF (vascular endothelial growth factor). VEGF has six isoforms (VEGF-A, -B, -C, -D, -E, and PLGF) that can bind to three tyrosine kinase receptors (VEGFR-1,-2, and-3) leading to a complex network of downstream signaling (39). Fiedler et al. showed that a large proportion of AML patients express high levels of VEGF, leading to both induction of angiogenesis and reduction of apoptosis in AML cells. Furthermore, VEGF induces an increase of GM-CSF secretion by endothelial cells, which is a known mitogen for AML cells (40).

Mesenchymal stromal cells (MSCs) are known to provide microenvironmental support for HSCs but also have the potential for differentiation to multiple lineages including osteoblasts, chondrocytes and adipocytes. These processes are highly regulated and disturbances are involved in oncogenic events. Battula et al. showed that AML cells induce osteoblastic but inhibit adipogenic differentiation of MSCs by bone morphogenetic protein (BMP) signaling (41). This shift leads to a pre-osteoblastic niche that enhances AML expansion. Additionally, Hanoun et al. showed that AML progression leads to a reduction of the sympathetic nervous system, which is critical for MSC quiescence osteoblast differentiation and even hematopoietic stem cells (16). This initiates an expansion of osteoblastic-primed MSCs, which can contribute to AML progression (17). Frisch et al. described that the secretion of CCL3 (also known as macrophage inflammatory protein 1α, MIP 1α) by AML cells leads to reduced levels of osteoblasts and osteocalcin in the blood. Inhibited osteoblastic cell function results in loss of mineralized bones and reduced healthy hematopoiesis (42). Taken together, these observations indicate that AML cells induce and require an osteoprogenitor-rich niche for their expansion, but block MSC differentiation to produce mature osteoblasts.

The role of adipocytes during leukemogenesis is also controversial. The existence of bone marrow adipocytes has been known for more than a century, but only recently has their function been deciphered. In contrast to the idea of silent fat storage, bone marrow adipocytes are highly active regulators of the bone marrow metabolism and the overall body homeostasis by secretion of adipokines (43). Adipocytes were identified as negative regulators for the HSC compartment (44) and Boyd et al. described the disruption of the adipocytic niche by AML (45). In contrast, Shafat et al. described a supportive effect of bone marrow adipocytes for the survival and proliferation of AML cells based on the transfer of fatty acids from adipocytes to blasts (46). In general, the amount of adipocytes in the bone marrow can fluctuate and react to several metabolic situations, such as starvation or cytotoxic stress. Since chemotherapy induces adipogenesis that leads to a different bioavailability of drugs in the bone marrow, the role of adipocytes for AML development and survival needs further investigations (47).

Although bone marrow fibroblasts are a major component of connective tissue, knowledge about the role for AML development is very limited. It is known that fibroblasts produce extracelluar matrix fibers but also regulate the microenvironment by cytokine and chemokine secretion (48). Furthermore, cancer-associated fibroblasts are considered to influence the chemo-resistance of many solid tumors. Zhai et al. presented that fibroblasts are increased in leukemic bone marrow and can provide chemo-protective elements for AML (49). Further animal experiments need to verify these findings.

The immunological microenvironment of leukemic cells plays another fundamental role for leukemic development and chemotherapy resistance (50). The normal bone marrow hosts various mature immune cell types, including T and B cells, dendritic cells, and macrophages. Usually, immune cells recognize abnormal cells on the basis of mutated proteins and are able to eliminate them (51). But AML cells can escape an immune response by creating an immunosuppressive microenvironment, where both innate and adaptive immune responses are dysregulated (50). It was shown that AML can reduce T and natural killer cell function and cytotoxicity and furthermore induce populations of regulatory T cells (Treg) through the activation of immune checkpoint markers like programmed death-1 (PD-1). These markers are crucial regulators of the immune response and play a central role for self-tolerance. Physiologically, ligands like PD-L1 are expressed on antigen-presenting cells and binding to their receptors results in the reduction of T-cell activation. Interestingly, AML cells are known to express PD-L1 to escape immune mediated eradication and PD-L1 expression levels correlate with AML progression (52). Furthermore, mesenchymal stromal cells have immune modulatory functions (53). And even though the exact mechanisms are not fully elucidated, indoleamine 2,3-dioxygenase 1 (IDO1) seems to play a key role. IDO1 expression can inhibit T-cell proliferation and modulate the function of major cell populations involved in innate and adaptive immune systems (50).

In addition to the cells of the adaptive immune system, mononuclear phagocytes contribute to the regulation of HSC populations (54). Macrophages were shown to regulate osteoblasts and the HSC mobilization in the endosteal niche (14). Interestingly, AML leads to an invasion of AML-associated macrophages into the bone marrow and spleen, supporting their progression.

One critical component for AML engraftment and progression is the adhesion to the microenvironment. Several adhesion molecules (e.g., VLA-4, E-selectin, and CD44) were deciphered as playing a relevant role for AML (29). VLA- 4 (very late antigen 4) a cell surface ligand for VCAM-1 (vascular cell adhesion molecule 1) presented on mesenchymal stromal cells is highly expressed on AML cells. Jacamo et al. showed that the interaction of VLA-4 and VCAM-1 activates prosurvival and proproliferative pathways in both the leukemia and stromal cells via the NF-κB pathway, leading to higher chemotherapy resistance (55). Interestingly, patients with VLA-4 negative AML have a generally favorable outcome (56). Mouse experiments using a VLA-4 specific antibody in combination with cytarabine therapy showed an improved outcome. The second axis of cell adhesion showing promising results for new treatment strategies is the interaction of the adhesion molecule CD44 and E-selectin. The latter is expressed by endothelial cells and is bound by CD44 that is broadly expressed on AML cells. Antagonizing E-selectin increases chemosensitivity of AML under treatment (57). Furthermore, Jin et al. showed that the activation of CD44 eradicates AML stem cells (58). Targeting the adhesion of AML cells interferes with their embedding in their protective niche and therefore influences survival.

Besides adhesion, AML cells are also regulated by soluble factors secreted by niche cells, like Notch, CCL3, TGF-β, or CXCL12 (59–61). We want to highlight CXCL12 (C-X-C motif chemokine 12), also known as stromal-cell derived factor 1 (SDF-1) (62). CXCL12 is secreted by mesenchymal stromal cells and induces chemotaxis in leukocytes by binding to its receptor CXCR4 (C-X-C chemokine receptor 4). In normal hematopoiesis this axis regulates leukocyte trafficking. Chemotherapy stress induces the expression of CXCR4 in AML cells, leading to increased resistance and survival. Inhibition of CXCR4 sensitizes AML to chemotherapy and increased therapy-related apoptosis. Another level of intercellular signaling involves the exchange of information via membranous structures, like exosomes and tunneling nanotubes (TNT). A recent publication from Kumar and colleagues presented that AML cells secrete exosomes to manipulate mesenchymal stromal cells (63). This signaling blocked osteolineage development and led to a leukemic niche that supported the leukemic development and survival. In leukemia, TNTs were first described by Polak and colleagues, showing that acute lymphoblastic leukemia cells manipulate the mesenchymal stromal cells to induce the secretion of prosurvival cytokines and chemotactic proteins, improving their capabilities for chemotherapy resistance (64).

However, since most data are based on *in vitro* experiments, the exact mechanisms leukemia cells use for modifying their microenvironment to generate a shelter against chemotherapy still remain largely unknown.

## Animal models for AML niche analysis

To understand how AML cells interact with their niches, it is crucial to know spatiotemporal cellular interactions and dynamics in the bone marrow. Quantitative analyses of bone marrow mononuclear cells by flow cytometry present a well-established technique and can deliver important hints of changes in cellularity but information about localization and cell-cell interactions get completely lost. Furthermore, extraction of structural cell types, such as endothelial or mesenchymal stromal cells for flow cytometry includes enzymatic tissue degradation steps, which can be inefficient and therefor significantly adulterate the data. Only imaging of native tissues enables the study of cellular interactions in the multicellular niche, without losing single components of the processed tissue.

One powerful model to study the development of the hematopoietic system *in vivo* is the transgenic zebrafish (*Danio rerio*) (65). Hematopoiesis is highly conserved between mammals and zebrafishes and live imaging in zebrafish embryos is a very well-established method for HSCs research. The benefits of this model are i.a. the high reproduction rates, enabling the generation of hundreds of embryos at the same time and external fertilizations, allowing the study of developing embryos without dissections. Additionally, the zebrafish embryo is small and transparent, allowing the scientist to perform live cell imaging by confocal microscopy in the complete living organism. Leonard Zon and coworkers utilized a transgenic zebrafish line to visualize the dynamic colonization of the caudal hematopoietic tissue (CHT), which is comparable to the mammalian fetal liver. Utilizing high-resolution live imaging they were able to visualize the first steps of colonization of the developing CHT. They described for the first time how endothelial cells remodel around single HSCs to form a stem cell pocket (66). However, the utility of zebrafishes for studying adult hematopoiesis and leukemogenesis is limited, since adult zebrafish hematopoiesis resides in the kidney marrow, which is structurally distinct to the bone marrow of mammals (67).

In the last decades murine models became the main mammalian model system for hematopoietic research. Their convenient maintenance and handling, as well as their small body size, short reproduction cycle, and the ability for genetic manipulations are prerequisites for a biomedical research tool. The development of immunodeficient mice strains like NSG (NOD scid gamma) enables xenografting of patient-derived cells without pre-conditioning of the mouse with radiation or chemotherapy, allowing studies about interactions between human hematopoietic cells and their microenvironment (68). One major contribution of the patient-derived xenograft (PDX) models to the AML field was the identification and characterization of leukemia initiating cells (LICs) (32). However, several signaling components of the mouse are incongruent with those of humans. In particular AML remains one of the hematologic malignancies with low engraftment performance in NSG mice (69). As a result, several attempts have been made to customize the bone marrow microenvironment to enable myeloid infiltration. One promising approach is the development of humanized mouse strains with transgenic expression of human cytokines like the NSG-SGM3 mouse strain (70). Effects on the bone marrow structure and the localization of LICs in these humanized mouse models are owing and need to be elucidated in the future.

## Niche analysis by imaging techniques

The first analysis of the HSC localization in murine bone marrow using confocal laser scanning microscopy was presented by Kiel et al. (24). The structural differences between the semisolid bone marrow and the hard bone surface is challenging for histological sectioning methods. Utilizing the CryoJane system they were able to transfer thin tissue sections to object slides, where histological staining can be performed. Alternatively Kawamoto developed a method for hard tissue sections, using an adhesive film to allow sectioning independent of special section equipment (71). Visualization of xenotransplanted AML stem cells in the bone marrow by confocal laser scanning microscopy revealed a preferential engraftment within the osteoblast-rich endosteal niche that was defined as a twelve cell deep zone, starting with the bone surface (72). To improve the study of cellular distribution inside two-dimensional (2D) sections the laser scanning cytometry (LSC) technology was developed. LSC is an automated cellular analyzer that allows *in situ* imaging and quantitative analysis of fluorescent labeled cells within thin tissue sections (73). LSC records precise spatial localization information of each fluorescent molecule within the section and generates a detailed map of the analyzed tissue. Nombela-Arrieta et al. used this technique to study the spatial distribution of HSCs in the bone marrow and defined distinct endosteal niches (25). However, these 2D histological studies of the bone marrow fail to present the complexity of the three-dimensional (3D) network of blood vessels, scaffolding structures and hematopoietic cells. The approach of serial sectioning and computational reconstruction of the tissue geometry might improve the informational outcome but remains challenging. Therefore, the establishment of protocols for 3D confocal microscopy of bone marrow samples was a crucial step for the elucidation of the HSC niche.

**Figure 2:**
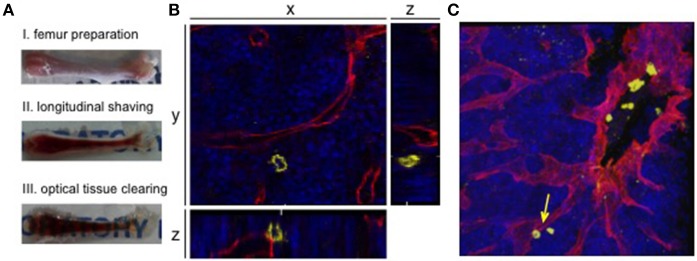
3D confocal visualization of leukemic cells in the murine bone marrow. **(A)** Whole-mount bone preparation of fixed cryopreserved femur. (I) Bone was cleaned from tissue for fixation and cryopreservation. Next it was shaved longitudinally from both sides on a cryostat until bone marrow was fully visible (II). After optical clearing tissue appeared transparent (III). **(B)** Orthogonal sections of a z-stack show the relevance of 3D visualization for niche analyses. The 2D plane shows a leukemic cell (CD45, yellow) without direct contact to a sinusoidal blood vessel (endoglin, red). Orthogonal sections show an adjacent blood vessel below the AML cell. DAPI signals mark the bone marrow tissue (blue). **(C)** Corresponding 3D z-stack image of bone marrow vasculature with engrafted human AML cells. Cell from orthogonal section is marked with an arrow.

Kunisaki et al. were first to present a protocol for whole-mount bone marrow confocal immunofluorescence imaging, revealing new information about the regulation of quiescence in HSCs (74). A milestone for the imaging of whole-mount samples was the development of tissue clearing substances. Like most tissues, bone marrow opacity undermines high-resolution microscopy due to light scattering caused by reflection off of molecules, membranes, organelles and cells (75). During the last years, several methods for optical tissue clearing were developed, including clearing via solvent- or aqueous-based clearing solutions. The simplest passive clearing technique uses immersion in a high refractive index solution, since this procedure has no influence on fluorescent molecules or tissue structures and can easily be implemented into running staining protocols. For details, we refer the reader to the review from Richardson and Lichtman (75). Utilizing optical tissue clearing Acar et al. were able to presented a 2015 study demonstrating the distribution of dividing and non-dividing HSCs in whole-mount bone marrow samples, including sophisticated methods for image annotation and analysis (76). The combination of second harmonic generation signals to visualize the hard bone structures and 3D visualization of blood vessels revealed that dividing and non-dividing HSCs locate in a persinusoidal niche distant from the endosteum. Since then, rapidly emerging deep-tissue imaging studies are reveiling bone marrow structures and improving the map of niche formation of hematopoietic and leukemic stem cells (Figure 2) (77–80). We want to highlight the work from Coutu et al., which provide an open source for imaging data to map nonhematopoietic cells and structures in the niche (77). The highly sophisticated analysis of the physical network of cellular interactions and localization within the bone marrow presents a comprehensive atlas of the distribution of bone marrow markers. It seems like the only limitations in this field are the availability of specific antibodies or transgenic mouse lines.

Scanning electron microscopy (SEM) is the most high-resolution imaging technique to visualize surfaces of cells and tissues in a high-resolution manner independent of available antibodies or transgenic mouse lines. The resolution is greater than with a light microscope but information are limited to the two-dimensional surface. However, there are several SEM techniques that can obtain 3D information about a sample, enabling the reconstruction of tissue structures. Serial block face (SBF-SEM) and focused ion beam scanning electron microscopy (FIB-SEM) both allow serial sections by ablating the specimen either with a diamond knife or a beam of ions with subsequent alignment of the data to allow reconstruction of a three-dimensional volume. Within this image individual structure can be segmented and qualitative and quantitative information can be obtained (81). FIB-SEM was used to characterize bone marrow adipocytes revealing highly dynamic cells with manifold interactions to its microenvironment (82). Identification of the cell of interest within electron microscopy is limiting this technique since it is solely dependent on visible structure hallmarks. This presents a particular challenge for xenografted AML cells, where the discrimination from to normal murine precursor cells is highly difficult.

In general, whole-mount imaging and computational modeling only allows visualization of snapshots of dynamic processes. The development of intravital microscopy solves this issue and has been used to study the behavior of HSCs and LICs in real time in the bone marrow. In 2005 Sipkins et al. presented two-photon microscopy data about the homing and engraftment of leukemic cells into the mouse calvarium bone marrow and described a specialized endothelial niche for acute lymphoblastic leukemia cells (15). This approach is minimally invasive and allows the visualization of living cells inside the bone marrow over hours. The development of a mouse calvarial window made of cover glass enables imaging even over weeks in multiple imaging sessions (83). Several groups have used and improved two-photon intravital imaging to study spatiotemporal distribution of cells in the calvarium and in the murine femur, but the latter remains highly invasive (84–89). The downside of intravital microscopy is a dependence on transplantation and transgenic models, as well as on injectable dyes (90). Since *in vivo* antibody staining is not sufficient for direct imaging, cells of interest have to be labeled either *ex vivo* before transplantation or transgenic reporter mouse strains have to be generated. In particular, *ex vivo* treatments can influence a broad range of cellular functions like localization, division and cell-cell interactions. It is for this reason that intravital microscopy has thusfar been unable to completely replace *ex vivo* confocal 3D imaging and vice versa. The future will show how well-researchers will be able to combine these techniques to visualize both a detailed and dynamic picture of the bone marrow niche of hematopoietic and leukemic stem cells.

## *In vitro* mimicking of the bone marrow niche

Visualizing cells within their niche *in vivo* is a sophisticated method to study leukemia-niche interactions. However, xenograft models never fully recapitulate the physiologic niche, since human cell engraftment requires the depletion of the immune system. Mice need genetic modification to deplete T-, B- and natural killer cells, and especially for AML a humanized microenvironment is necessary for the engraftment and development of the disease *in vivo*. But genetic engineering of mice is time and resource consuming and alternative models to study niche related pathways are required. One possibility to generate human-mouse chimeric bone marrow tissues is the usage of ossicle systems (91). These bony structures formed in ectopic regions show formation of bone with mature, vascularized bone marrow tissue. Since scaffold materials can be seeded with mesenchymal stromal cells or/and endothelial cells before implantation, ossicles are a useful tool to test genetic modification of niche cells in regard of stem cell and leukemic engraftment. The newest developments in the field of 3D printing have improved the types of ossicles that can be generated, including variable pore size and structures, usage of biomaterials or even the incorporation of growth factors into the scaffold (92). However, the native bone marrow is a highly complex tissue and these engineered scaffolds will still represent only a model with specific limitations.

Nowadays, there is a desire to reduce mouse models and replace them by reliable *in vitro* assays. But classical 2D coculture models are not sufficient to mimic the *in vivo* situation of the bone marrow niche. In addition to solid compounds, the bone marrow is regulated by the concentration of soluble factors, oxygen levels, availability of nutrients and even the flow of blood and liquids inside the niche, which applies mechanical stress. Utilizing biomaterials or bioreactors to generate 3D cell cultures will allow modeling the niche situation more closely. Either inorganic biomaterials like hydroxyapatite or hydrogels are successfully used to culture HSCs. State-of-the-art techniques like perfused 3D bone marrow analogs by Rödling et al., or the sophisticated bone marrow-on-a-chip by Torisawa et al. are probably the most advanced *in vitro* models to date (93, 94). Bray et al. used a 3D tri-culture of endothelial, mesenchymal stromal and AML cells to study drug resistance *in vitro*. Interestingly, they were able to show a greater drug resistance in the 3D model compared to a 2D coculture, underlining the relevance of niche mimicking models (95). All of these models provide an important *ex vivo* alternative to mouse models. Genetic and pharmacologic manipulations, as well as stimulation with cytokines can be performed in a direct and multimodal manner, opening the possibility of personalized drug response screens for therapy prediction.

## Vascularity in the bone marrow

The development of microscopy techniques to visualize the bone marrow niche under leukemic engraftment revealed not only changes on the molecular level but also in the overall structure, especially for the vascular system. In general, the bone marrow vascularity is responsible for the nutrient and metabolite turnover, oxygenation of the tissue and the ingress and egress of cells, like freshly differentiated immune cells. Blood vessels are made up of several different cell types. The inner layer is composed of endothelial cells (ECs), which is covered by perivascular cells (pericytes). These pericytes are embedded in the subendothelial basement membrane and connect ECs with smooth muscle cells, which generally cover large vessels, like arteries and veins (96). Remarkably, bone marrow vessels are mainly formed by a sinusoidal network consisting of a single layer of ECs (97). As in other organs, the vasculature of the bone marrow is organized in a specific order. The arteries are longitudinally aligned along the diaphysis of long bones and infiltrate into the bone marrow via branching to small arteriols and finally to a capillary network with larger diameter. To exit the bone marrow, capillaries drain together into a large central vein, which passes the hard, calcified bone matrix going back to the periphery (98).

Adams and coworkers defined two subtypes of bone capillaries, so called H and L that are distinguishable by function, surface marker expression, and structure (96, 99). Type H capillaries express high levels of CD31 and endomucin and are located in the metaphysis, which is the region of the bone containing the avascular growth plate. They are surrounded by osteoprogenitors, connected to arteriols and play an important role in angiogenesis. In contrast, type L vessels express only low levels of CD31 and endomucin and lack arteriolar connection and osteoprogenitor association but are surrounded by leptin receptor (LEPR)^+^ and CAR cells. They form the dense and highly branched capillary network of sinusoids in the bone marrow cavity that is known to regulate the HSC compartment (13, 18). Blood flows first through the arteries into type H capillaries before it enters the more permeable sinusoidal type L network at the interface between metaphysis and diaphysis. This leads to metabolically distinct environments, with a well-oxygenated metaphysis and a highly hypoxic diaphysis, due to the lack of direct arterial supply (99, 100).

Several studies showed that hypoxia is a key regulator of the niche-mediated dormancy and maintenance of HSCs (101, 102). Low oxygen supply has direct effects on the metabolism of a cell, changing it from oxidative phosphorylation to cytoplasmic glycolysis (103). The latter is less efficient in overall energy production and pushes cells into energy preserving dormancy. A benefit of shutting down oxidative phosphorylation are the reduced rates of intracellular reactive oxygen species (ROS) production (104). ROS management is crucial to keep HSCs in a quiescent state, since excessive levels of ROS can induce oxidative stress leading to protein, lipid, and DNA damage. Therefore, it is not surprising that high levels of ROS are linked to AML development by inducing genomic instability leading to chromosomal deletions or translocations (105). Itkins et al. showed that permeable sinusoids display higher ROS levels, lower blood flow, and shear rates compared to less permeable arterial blood vessels (106). Hematopoietic cell rolling and adhesion events as well as transendothelial migration of mature leukocytes and immature HSC occurred exclusively via sinusoids. Most recently, Passaro and colleagues showed that engraftment of patient-derived xenograft AML samples (PDXs) into mice increased the vascular permeability compared to control mice engrafted with healthy human HSCs (85). Transcriptome analysis of vascular ECs upon human AML engraftment showed that the NOX4-NOS3-axis (NADPH oxidase-nitric oxide synthase 3) is highly activated, a pathway that is known to act in response to stress induced by hypoxic conditions and to induce vascular leakiness. Accordingly, the bone marrow of xenografted mice showed increased levels of NO (nitric oxide), an inducer of vascular permeability; keeping in mind that increased vascular leakiness is associated to poor drug delivery, favoring resistance to therapy and relapse (107). Remarkably, the pharmacological inhibition of NO production in combination with cytarabine reduced AML progression and restored normal vasculature in PDX models, pointing toward a potential clinical strategy for NOS inhibition in the bone marrow vascular niche in AML. Taken together, these observations indicate that the bone marrow contains several distinct vascular sites, with defined functions and structures that regulate the fate of HSC and might have specific functions for leukemia inducing cells.

## The vessels and angiogenesis in AML

Angiogenesis is the process of blood vessel development from pre-existing ones and is an absolute requirement for the viability and growth of solid tumors (108). The tumor-angiogenic process is mostly initiated by pro-angiogenic factors derived from tumor cells, which activate the initiation of irregular and uncontrolled vascular growth (109). Newly formed tumor vessels appear abnormal and leaky, affecting the local blood flow, metabolite exchange, and oxygenation. These aberrant structures influence drug delivery, tumor growth, metastatic potentials and ultimately the survival probability for a patient (110). Since AML is considered a liquid tumor without a compact structure, angiogenesis in AML was initially underestimated. De facto, leukemia cells, like tumor cells, are highly dependent on angiogenesis in the bone marrow and AML is associated with an increase in bone marrow microvascular density (MVD) (111, 112). More recently, clinical data has proven that bone marrow biopsies of AML patients show an increased number of sinusoidal blood vessels in comparison to healthy individuals that corrects to normal after leukemia remission. Additionally, the degree of MVD can be used as a prognostic marker, since higher MVD correlates with an increased risk of relapse and a shorter overall survival (113).

## Antiangiogenic therapy of AML

Since AML is strongly connected to the vascular niche and remodeling of bone marrow vascularization, antiangiogenic therapy seems to be a logical clinical strategy. To date, several drugs with antiangiogenic properties had been tested in clinical studies, but clinical outcomes have been disappointing (29, 114). One prominent example is bevacizumab, a monoclonal anti-VEGF antibody that has the potential to decrease VEGF levels in bone marrow. But clinical studies showed that AML progression remain unchanged (115). Only the combination of bevacizumab with standard induction chemotherapy showed a slight improvement in patient outcome (116). Since VEGF signals via receptor tyrosine kinases (RTKs) with the intracellular signaling cascade, TKIs (tyrosine kinase inhibitors) are a family of small molecules that are under clinical investigation for AML treatment with relevance to angiogenesis (e.g., semaxanib, sunitinib, or sorafenib). Interestingly, some of these drugs block other RTKs as well, broadening the spectrum of their efficacy. For example semaxanib was one of the first TKIs in a clinical trial that targets VEGFRs, but also inhibited stem cell factor receptor (KIT) and FLT3 (FMS-like tyrosine kinase 3) (117). Application of semaxanib was generally well-tolerated, but the effect on leukemic progression was absent, so further clinical development was discontinued. Comparable to semaxanib, sunitinib binds to a broad range of RTKs and is approved for the treatment of gastrointestinal stromal tumors. However, for AML treatment, sunitinib showed only modest clinical activity (118). Sorafenib is a potent inhibitor of VEGFR, KIT, PDGFR (platelet-derived growth factor receptor) and FLT3; the latter of which is mutated in 25–30% of AML cases, causing a constitutively active variant and promoting leukemogenesis (119). Clinical data showed that sorafenib reduced leukemic growth from AML patients with FLT3 mutations but not in patients without mutations. Beside its specific effect in AML carrying FLT3 mutations, clinical activity is only modest and in combination with standard chemotherapy sorafenib showed no additional benefit. Lenalidomide is another example of a VEGF inhibitor. This thalidomide derivative is an antiangiogenic and immunomodulatory drug with a decreasing effect on VEGF expression. However, clinical relevance for AML treatment is controversially discussed (120, 121).

In addition to VEGF, another major class of endothelium-specific growth factors includes the members of the angiopoietin family (Ang1, 2, 3, and 4). Interestingly, high expression of the pro-angiogenic factor Ang2 is an independent prognostic factor for overall survival in AML (122). However, clinical studies with trebananib, a neutralizing peptibody against Ang1 and Ang2 showed only a limited clinical efficacy (123). Overall, clinical data indicates that monotherapy with antiangiogenic drugs lack the efficacy to treat aggressive leukemia's, only the combination of antiangiogenic drugs with standard chemotherapy is likely to have a benefit for patient's outcome and need more clinical investigations.

An alternative approach may be to specifically harm the perivascular niche and the AML support by ECs with vascular disrupting agents (124). Combrestatins cause a breakdown of the vascular architecture by targeting the microtubule of rapidly proliferating EC. Clinical trials are ongoing to elucidate the effect of vascular disrupting agents on the progression of AML (125). In contrast, Duarte et al. found that the loss of bone marrow blood vessels can be associated with increased chemotherapy resistance of AML cells (84). In detail, AML progression leads to differential remodeling of the vasculature into a leukemia-preferential microenvironment. Dependent on their anatomical localization in the bone marrow, blood vessels were either obliterated (endosteal type H capillaries) or rescued but modified (central marrow vessels). Endosteal located AML cells are enriched in inflammatory and TNF gene signatures and express higher levels of CXCL2. Both, TNF and CXCL2 are known factors for vascular destruction and angiogenesis inhibition (126, 127). The consequences of this remodeling are the loss of HSCs specifically from endosteal areas and survival of leukemia cells under chemotherapy. Interestingly, induction of Notch signaling in ECs leads to increased numbers of endosteal vessels and furthermore to a decrease of chemotherapy resistant AML cells in the bone marrow, leading to the hypothesis that rescuing endosteal vessels before chemotherapy may actually improve the outcome and maybe the overall survival of patients, although clinical studies are needed to confirm this mechanism (84).

## Other therapeutic targets in the niche

Besides antiantiogenic approaches, other known niche components are under evaluation for targeted therapy. Developing CXCR4 inhibitors has made the most progress. The rational behind the blocking of this signaling pathway is to mobilize AML cells out of their protective niche to make them available for chemotherapy. Strong clinical evidence was shown in phase I studies for plerixafor (AMD3100), a small molecule inhibitor of CXCR4 that increased the rate of complete remissions in combination with cytarabine and daunorubicin but adverse events were frequent (128). Other CXCR4 inhibitors like ulocuplumab and BL-8040 present additional pro-apoptotic effects on AML cells and showed in combination with induction therapy accelerated mobilization and improved outcome for the patients (129). Finally, CX-01, a anticoagulant, binds CXCL12 with a high affinity, effectively blocking CXCL12/CXCR4 signaling. Combination with intensive therapy was well-tolerated by patients and showed enhanced treatment efficacy (130). With such promising preliminary results using CXCR4 inhibitors further clinical trials will likely follow to further elucidate the therapeutic potential of this axis.

The interaction between VLA-4 on AML cells and VCAM-1 on mesenchymal stromal cells represent another promising candidate for targeted therapy. Natalizumab was the first humanized VLA-4 monoclonal antibody tested in a xenograft AML model. Despite its beneficial effects on overall survival, its utility is limited since it can induce leucoencephalopathy (131, 132). Another pre-clinical VLA-4 inhibitor is AS101 which decreases the pro-survival PI3K/Akt/Bcl2 signaling to sensitize AML cells to chemotherapy. A mouse xenograft AML model showed that AS101 can abrogate drug resistance of leukemic cells and prolong survival in mice after chemotherapy (133).

Last, the therapeutic potential of the inhibition of E-selecting binding to CD44 should be noted. H90, an activating monoclonal antibody against CD44 and GMI-1271, a specific small molecule inhibitor of E-selectin, both showed a reduction of the leukemic burden in xenograft AML models (58, 134). These preclinical results would justify further explorations to elucidate the therapeutic potential of these inhibitors for AML treatment in combination with chemotherapy. The availability of a FDA-approved CD44 monoclonal antibody, bivatuzumab, that is currently used for clinical trials in solid tumors could potentially allow a novel clinical trial in AML patients.

## Immunotherapy

The immunosuppressive role of AML cells has a strong impact on chemotherapy survival and the causative mechanisms present a promising target for therapy. One approach targets the immune checkpoint molecules expressed by AML cells. Several immune checkpoints are initiated by ligand-receptor-interactions and therefore can be blocked with antibodies or blocking fragments of the receptor or ligand (135). Unfortunately, an initial clinical trial with the monoclonal PD-1 antibody CT-011 showed disappointing results for AML patients (136). Another approach for immunotherapy is the treatment with bispecific T-cell engager (BiTE®) constructs. These recombinant antibodies include two variable domains to bind to two different antigens at the same time (137). One domain binds CD3, a component of the T-cell receptor complex, to connect T-cells to tumor cells initiating the MHC-I independent cytotoxic synapse leading to the lysis of the tumor cell. The first BiTE® for clinical application is blinatumomab, a combination of CD3 and CD19 for therapy of a specific form of acute lymphoid leukemia. For AML therapy AMG 330, a CD33/CD3-BiTE® is in a clinical phase (138). Interestingly, although being independent of MHC-I-interactions, the effectivity of AMG 330 has been shown to be dependent of costimulatory and coinhibitory T cell ligands (139). Moreover, treatment with AMG 330 led to an upregulation of the immune checkpoint molecule PD-L1 on AML cells and blocking of the PD-1/PD-L1 interaction enhanced AMG 330 mediated cell lysis (140). In this regard, the TIGIT-PVR/PVRL2 immune checkpoint axis recently emerged as a promising target for AML therapy (141). Our lab could thereby show that the blocking of the inhibitory immunoreceptor TIGIT or the corresponding ligands poliovirus receptor (PVR, CD155) and poliovirus receptor-related 2 (PVRL2, CD112) augment the antileukemic effects of AMG 330 (141).

Lastly, we want to mention the promising approaches with autologous or allogeneic T cells engineered with synthetic chimeric antigen receptors (CARs) (142). The artificial T cell receptors are designed to specifically target tumor cells, with binding inducing T-cell activation, differentiation, inflammation, and targeted cytolytic killing of the target cancer cells. For acute lymphoblastic leukemia (ALL) two CD19 CAR T cell products, tisagenlecleucel and axicabtagene ciloleucel, were approved for clinical usage in the United States. Unfortunately, AML cells lack ideal target antigens, since most surface proteins are expressed on normal myeloid precursor cells as well, and unlike B-cells patients cannot survive without functional myeloid cells. But given the remarkable cure rates for relapsed B-ALL, several preclinical trials are ongoing to identify a potent target for CAR T cell therapy of AML. Details were reviewed recently by Tasian (142). Here, we want to highlight CLL-1 (C-type lectin-like molecule-1) that is expressed on the majority of AML blasts and on leukemia initiating cells but not on hematopoietic stem cells (143). Recently Dr. Fang Liu (Chengdu Military Hospital, Chengdu, China) demonstrated that simultaneously targeting of multiple antigens like CD33 and CLL-1 is an effective strategy to eliminate AML. However, disregarding the promising results of immunotherapy for AML patients, only limited information are present about the effects of immune cells in the bone marrow. Future work will be needed to elucidate how immune cells reach the AML cells in their niches and how the microenvironment influences the outcome of the therapy. Visualization of the effects of immunotherapy to the bone marrow niche is a central task to understand and improve this approach of therapy.

## Conclusions

There is an ever-growing number of targeted therapeutic strategies to treat acute myeloid leukemia, yet patient outcome remains poor and clinicians have an urgent need to receive new treatment strategies for patients. This result is likely due to the highly heterogeneous and highly polyclonal nature of AML and approaches that target specific mutations in AML cells may result in the eradication only of single subclones and therefor are inadequate to rid patients of the disease. Because complex interactions with the bone marrow microenvironment influence the survival and progression of AML, research is now focused on deciphering the bone marrow niche composition to identify new drugable targets. These interactions are less clone-specific and therefore represent a promising strategy to overcome the obstacles of individual cell heterogeneity. Furthermore, residual LICs are thought to drive disease relapse and targeting their sheltering niche might offer a means to improve eradication of AML. Unfortunately, blocking signaling pathways also harbors the risk of unpredictable side effects and toxicity. Most pre-clinical research is done in animal models to study drug effects on disease progression, and there are important limitations in translation of animal model results to human patients. Thus, researchers should seek to find good *in vitro* models that predict clinical drug responses. There is a strong demand for new agents with good tolerance in a broad range of AML patients, and increased efficacy relative to conventional chemotherapy. Any possibility to replace one or more components of treatment regimens in frail patients to reduce long-term effects on the maintenance of healthy HSCs would have significant clinical benefit. Technical developments of the past few years have yielded significant progress for understanding the physiological constitution of normal HSC niches and the malignant alterations that drive AML progression. However, the regulation of AML cells in their niche is highly complex and we have just started to understand the tremendous potential of targeting the bone marrow microenvironment to eradicate AML cells. Ongoing clinical studies and upcoming research results will point out new avenues of treatments, giving the hope that AML will 1 day become a treatable disease.

## Author contributions

LB wrote the manuscript. WF and JW contributed to the writing and editing of this manuscript.

### Conflict of interest statement

The authors declare that the research was conducted in the absence of any commercial or financial relationships that could be construed as a potential conflict of interest.
